# Prior Distribution and Entropy in Computer Adaptive Testing Ability Estimation through MAP or EAP

**DOI:** 10.3390/e25010050

**Published:** 2022-12-27

**Authors:** Joel Suárez-Cansino, Virgilio López-Morales, Luis Roberto Morales-Manilla, Adrián Alberto-Rodríguez, Julio César Ramos-Fernández

**Affiliations:** 1 Basic Sciences and Engineering Institute, Systems and Information Technologies Research Center, Intelligent Computing Research Group, Autonomous University of Hidalgo State, Col. Carboneras, Mineral de la Reforma 42184, Hidalgo, Mexico; 2 Department of Software Development, Advanced Computing and Innovation Research Group, Polytechnic University of Tulancingo, Tulancingo 43629, Hidalgo, Mexico; 3 Department of Mechatronics, Smart Technologies Applied to Social Development Research Group, Polytechnic University of Pachuca, Zempoala 43830, Hidalgo, Mexico

**Keywords:** entropy, CAT, Kullback–Leibler divergence, likelihood function, maximum a posteriori, expectation a posteriori, Bayesian inference, performance index function, item response theory, item characteristic curve

## Abstract

To derive a latent trait (for instance *ability*) in a computer adaptive testing (CAT) framework, the obtained results from a model must have a direct relationship to the examinees’ response to a set of items presented. The set of items is previously calibrated to decide which item to present to the examinee in the next evaluation question. Some useful models are more naturally based on conditional probability in order to involve previously obtained hits/misses. In this paper, we integrate an experimental part, obtaining the information related to the examinee’s academic performance, with a theoretical contribution of maximum entropy. Some academic performance index functions are built to support the experimental part and then explain under what conditions one can use constrained prior distributions. Additionally, we highlight that heuristic prior distributions might not properly work in all likely cases, and when to use personalized prior distributions instead. Finally, the inclusion of the performance index functions, arising from current experimental studies and historical records, are integrated into a theoretical part based on entropy maximization and its relationship with a CAT process.

## 1. Introduction

When one wants to explain some relationship between latent traits of individuals, for instance, unobservable characteristics or attributes and their manifestations (observed outcomes, responses, or performance), then item response theory (IRT) becomes a valuable formal tool. IRT is a family of models to analyze and predict the behavior of the involved variables, and their applications cover different assessment scenarios. Item exposure control, item calibration, and automatic item generation are only some examples of these scenarios and the involved variables. From a theoretical point of view, there are several well-known models to analyze these topics and potentially helpful tools to propose novelty solutions [1,2,3,4,5]. These models consider a set of items to define a measurement instrument. One set of parameters specifies the item’s characteristics that depend on the particular application. The results of the application of the measurement instrument provide information about the examinee’s latent trait. IRT assumes that the latent construct values (e.g., stress, knowledge, attitudes values) and some items’ parameter values are organized in an unobservable continuum as random variables. Thus, IRT helps to establish the position or value of the examinee’s latent trait on that continuum by considering the items’ characteristics and the quality of responses to them [6,7].

Items with different presentation and answer formats, examinees, and examiners are just a part of the assessment scenario. Particularly, computer adaptive testing (CAT) is an area where the application of IRT is highly useful to automatize performance assessments.

In general, IRT and its application to CAT assume the existence of a pool of items that by construction has finite cardinality. The CAT process supposes that, through experimentation, the pool contains calibrated items (calibrated items pool or CIP), i.e., a previous experiment provides information about the values of the items’ parameters that define its corresponding characteristics.

The item calibration process entails a statistical analysis of the responses arising from a set of test subjects, and the fitting of the experimental data to a sigmoidal cumulative distribution function (CDF) model assigned to every item. This procedure defines the corresponding item’s parameters and, therefore, the item characteristic curve (ICC) which depends on the latent trait as *ability*. The parameters of an ICC have an interpretation in terms of the item’s difficulty and the item’s discrimination capability, among others, and they influence the determination of the value of the latent trait under analysis. There are some well-known ICC models, such as 1PL, 2PL, and 3PL, although one can find 4PL and 5PL models, too. Naturally, 1PL (one-parameter logistic model) and 2PL (two-parameter logistic model) are the simplest models since their parameters have a direct interpretation and their relationships with the process of searching for the latent trait are clear.

Equation (1) defines the general structure for the 2PL model and gives the conditional probability of correctly answering an item with known difficulty and discriminant:

(1) $$P\left(\theta |\mu ,\alpha \right)=\frac{1}{1+e^{-\alpha (\theta -\mu )}},$$

where the parameters μ and α represent, respectively, the item’s difficulty and the item’s discriminant, and θ refers to the examinee’s ability.

An examinee participating in a CAT has to pass through three steps.

1. Assignment of an initial estimate of the examinee’s ability or the item’s difficulty, since the system needs to know the characteristics of the first item in the evaluation process.
2. The system saves the examinee’s response, decides if the examinee gave a correct answer or not, and builds the response pattern for this specific testing process.
3. The system considers the response pattern and the selected latent trait model to build a likelihood function, intending to decide what proper item (calibrated) must come next. There are several methods to do this, and here we apply the definition of the prior distribution. After deciding what item comes next, the CAT procedure poses this item to the examinee, and the testing returns to the second step again. Our main contribution aims to solve some problems in this step.

A reliable estimate of the next question to be presented, for instance, through the concept of the maximum likelihood function, requires at least two responses to the presented items in the evaluation process. One of the items needs to have a right answer, and the other an incorrect one. Only in this case, the likelihood function will have an extreme point in the set of values of abilities and, therefore, a maximum value at this point.

Note that in the event that all the answers obtained were correct (or incorrect), the  likelihood function is just a sigmoid (the ICC of the items) that does not have extreme points in the domain of the examinee’s ability. Because of this reason, it becomes impossible to compute the next estimation of the examinee’s ability.

To overcome the difficulty of estimating the next examinee’s ability by just using the likelihood function, some authors have proposed different options:

1. The use of two fictitious items with high and low probabilities to ensure that the examinee answers alternatively correctly and incorrectly to the items.
2. The use of heuristic formulas to estimate the examinee’s latent trait until a maximum likelihood makes it possible to estimate the value.
3. To define prior distributions until one can apply a likelihood function to estimate the examinee’s ability. This proposal relates directly to the statement of the research problem in this paper.

The first option has the inconvenience that the estimated latent trait value after applying the first non-fictitious item reaches very extreme values, and the second non-fictitious item of the CAT process provides more information for that extreme value of ability. Thus, the second non-fictitious item becomes less informative for the final ability value and it does not contribute considerably to the test precision [8,9].

The second option has the inconvenience that in some circumstances the CAT process does not converge, although when the increment (or decrement) of the latent trait value is variable this phenomenon does not occur [8,9].

Finally, the third option has several inconveniences:

(i) A general use of prior information in educational assessment appears to be inhibited solely by the assumption that including a priori information on test scores in performance assessment may be unfair to students [10].
(ii) Assuming that regular evaluation practices include information provided by the examinee (regarding past experiences) or collected from multiple sources in the assessment procedure without specifying the type of the sources [10].

Additionally, there is not much information about the potential risks when prior information is not perfectly accurate. Overconfidence in inaccurate prior information may in fact increase test length and/or lead to severely biased final latent trait estimates. In this event, then the system could, for example, select an incorrect starting point or introduce bias in the trait estimation process, and provide items that do not match the participant’s trait level [10]. On the other hand, the level trait does not depend solely on the examinee’s performance but on the values of mean and variance that one assigns to the trait’s prior distribution in the population [8].

From a theoretical point of view, depending on the established a priori distribution, one can obtain a multimodal posterior so that the Bayesian MAP estimation might refer to a local maximum [8].

Finally, in some cases, the Bayesian procedures provide estimation with a specific regression toward the mean of the prior distribution of the latent trait. This phenomenon can favor examinees with low levels and affect examinees with high ability [8].

There are several advantages and drawbacks of introducing information before starting an adaptable evaluation process. The usual way of building prior distributions lends itself to subjectivities, even though the benefits in the administration of the evaluation are undoubted [11,12]. However, the subjectivity inherent in the prior distribution can be minimized as long as reasonable evidence supports the distribution proposal [11].

In this work, we address the role that the entropy can play in the reduction of this subjectivity in the construction of the prior distribution by using a set of proposed constraints related to entropy. Any distribution must satisfy these constraints that consider, for instance, its first and second moments and the academic framework such as, for example, school dropout and failure, among others.

### 1.1. Problem Statement

The use of Bayesian statistical inference in the CAT process is delicate and has to justify the application of essential components such as the prior distribution [13]. Different theoretical and experimental techniques exist to determine the prior distribution to initialize the CAT process. Some authors suggest that physical, mathematical, engineering, expert opinion models, historical data under similar circumstances, or other reasonable information can support the prior proposal [13]. Thus, we formally introduce the models related to academic performance, for example, the failure rate, the dropout rate, the study habits index, and the subject comprehension index, among others, to further specify the structure of the prior distribution by using the concept of entropy.

#### 1.1.1. Preliminaries

The estimation of the ability of a test subject presents problems at the beginning of the evaluation process when using the maximum likelihood method and when the examinee responds correctly or incorrectly to all the test items. Several proposals solving this problem have been published and there are some options based on Bayesian inference [14]. In particular, the MAP or EAP techniques use the concept of the prior distribution, with the drawback that the definition of the structure of this distribution can lead to subjectivities.

#### 1.1.2. Originality

Within the given context, there is not enough information about the best prior distribution to be selected. Due to the Bayesian nature, MAP or EAP techniques require previous knowledge of the prior distribution, which contains initial statistical information about the ability of the examined subject.

One typically uses a normal distribution [15,16,17], but there is no evidence that this is necessarily correct since there is no reliable way to support the decision to opt for one prior type of distribution over another. The initial choice of the a priori distribution is paramount since it directly affects the calculation of the skill estimate and other parameters.

Furthermore, the structure of the psychometric model supporting the Bayesian inference process must be considered. An adequate structure selection provides an appropriate interpretation of each item’s characteristics, predicts the consequences of using a psychometric model with the selected characteristics, and ensures the relationship between these options and the multimodality and bias characteristics in the a posteriori distribution that finally helps to estimate the corresponding latent trait [18,19,20,21].

#### 1.1.3. Impact

In order to solve the former problems, one must then propose the form of the prior distribution through formal criteria to select good prior distributions. Some authors define some non-formal criteria and give quite illustrative examples of how the selection of a priori distribution affects the posterior distribution [22,23]. However, this research paper works mainly with the concept of entropy and, in a first instance, with the definition proposed by Shannon [24].

### 1.2. Article Structure

The paper is organized as follows: Section 2 focuses on a short hypothesis or conjecture statement and the paper’s objectives. Section 3 contains a brief discussion about some works on the importance of the prior distribution and the most common assumptions that the researchers make on its structure. This part also discusses the role that entropy could play in determining the *a priori* distribution and the previous work in this regard, but not within the framework of a CAT.

Section 4 briefly describes the differences between the 1PL and 2PL latent trait models and explains the meanings of the difficulty and discriminant parameters. Through these models and definitions, the concepts of maximization a posteriori, or MAP, and expectation a posteriori, or EAP, and their relationships with the prior distribution are introduced.

In addition, one recalls Shannon’s concept of entropy and states the ansatz (assumptions about the form of an unknown function, made to facilitate the solution of a problem) that give rise to the proposed method for estimating the prior distribution. Section 5 illustrates our numerical experimentation results, and provides and discusses the findings about the structures of the a priori distributions obtained through the proposed method. Finally, Section 6 synthesizes the results from numerical experiments and remarks some comments about the future work within the topic of the paper.

## 2. Hypothesis or Conjecture Statement

The specification of the prior distribution is a problem that does not have a straightforward solution in a CAT process. Part of this is due to the lack of formal procedures to get an analytical form of the distribution since there is no standard procedure on how the required information, to start the CAT process, can be integrated into a methodology to get an approximation to the model defining acceptable prior distributions.

### 2.1. Hypothesis Statement

If there is no formal procedure to determine prior distributions to initialize the CAT process, and Shannon’s entropy plays the role of the objective function depending on the *a priori* information distribution, which is subject to constraints of normality, mean values, and variance of the ability, in addition to the satisfaction of academic performance constraints considering failure, study habits, subject comprehension, and dropout rates of the course of interest, among others, then the formal finding of a prior distribution to initialize a CAT process is possible.

### 2.2. Objectives

Our general objective is to build informative prior distribution functions by considering the maximization of Shannon’s entropy as a cost function that depends on the distribution of *a priori* information, subject to normality, mean, variance, and academic performance constraints, to obtain formal prior distribution expressions. The specific objectives are the following:

1. To propose an ansatz about the school performance of the examinees, considering that they must be random functions depending on the random variable defined by the latent trait θ and some specific parameters, through the analysis of qualitative results obtained by various authors and, with these results, subsequently introduce distribution constraints based on the proposed assumptions.
2. To build an objective function to maximize entropy by considering the definition of entropy and the proposed ansatz in objective 1, and to obtain a methodology building and applying prior distributions in the CAT process.
3. To obtain experimental results numerically by simulating the behavior of the CAT process, to later make comparisons of the advantages and disadvantages of different scenarios that use prior distribution estimations.

## 3. State of the Art

The determination of the a priori distribution is experimental or through consultation with experts. However, regarding the role that entropy can play in searching for an adequate prior distribution, one can find a few research works on the topic. In this sense, to know how the *a priori* distribution behaves, one needs prior knowledge of the properties that it may have (the normality of the distribution is the simplest example of this knowledge, but there may be some other properties that are possible to know beforehand) [25,26].

The concept of prior distribution plays a fundamental role in Bayesian inference, so experimental determination of how to obtain these distributions and what theoretical methods should be to get something similar are paramount. To build a prior distribution, it is first necessary to specify representative random variables. In this sense, there are several possibilities that this paper introduces.

In the first instance, one assumes that the prior distributions must be related to the parameters of the selected psychometric model and the examinee’s latent trait variable to evaluate as proposed in [27,28,29,30] through the experimental construction of the corresponding prior distributions.

Additionally, one can consult experts in the knowledge domain to evaluate in order to obtain an opinion about the form or structure that the a priori distribution should have [28,30].

Despite not being connected to CAT systems, one can find in the literature some theoretical attempts to determine the structure of the prior distribution using the concept of entropy [26]. In addition to possibly getting the expert’s opinion, no known procedure integrates the results of the experimental process which, with a theoretical basis, can specify the characteristics or conditions under which one can obtain adequate prior distributions; that is, leading to unbiased posterior distributions, without multimodality, and to reliable latent trait estimates [18,19,20,21].

From a theoretical point of view, some contributions have dealt with the topic of informative and non-informative prior distributions, and they apply these definitions as academic examples to show the effects that the *a priori* distribution has over the *a posteriori* distribution [31].

In practice, heuristic distribution applications are analyzed when they are not supported by experimental data. In fact, some authors state that the practical consequences of using a prior distribution can depend on data. A heuristic distribution, such as the uniform or the normal with zero mean and unitary variance, can lead to nonsense inferences even when it has a large sample size. Currently, the study of prior distributions becomes relevant to analyze problems inside the frontiers of applied statistics [32,33].

In this sense, our paper integrates the experimental part of obtaining information related to the examinee’s academic performance into the theory of maximum entropy. The structure of the academic performance index functions supports this experimental part, which, as an additional result, explains under what conditions one can use heuristics priors. Additionally, the paper remarks that the heuristic prior distributions could not properly work in all the cases and that one must consider personalized prior distributions instead. Finally, the inclusion of the performance index functions, arising from current experimental studies and historical records, are integrated into a theoretical part based on the entropy maximization and its relationship with a CAT process.

## 4. Modeling Initialization of the Evaluation Process

**Figure 1:**
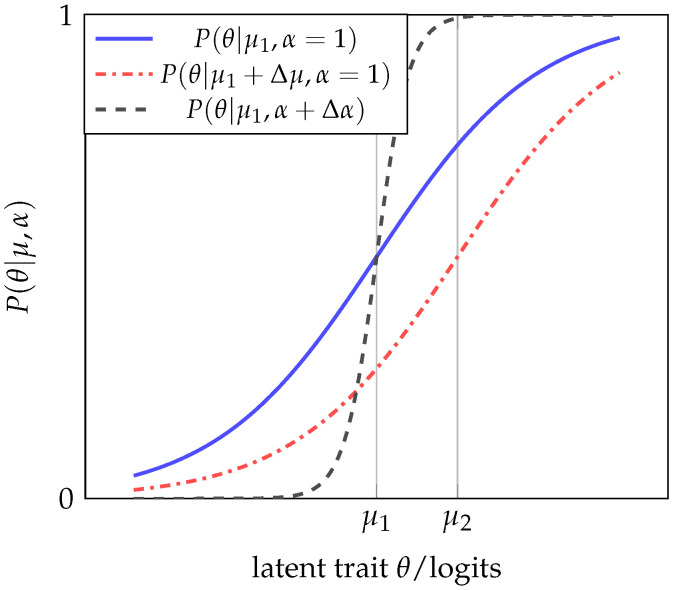
Effect of the difficulty value μ and the discriminant value α of an ICC in the case of the 1PL and 2PL latent trait models.

Geometrically, the characteristic curve of an item with difficulty $\mu_{1}$ differs from another associated with an item with difficulty $\mu_{2}$ by a simple shift to the left or right on the domain of the ICC, which is given by the latent trait values, depending on whether $\mu_{1}<\mu_{2}$ or $\mu_{2}<\mu_{1}$, respectively, as shown in Figure 1. On the other hand, the 2PL latent trait model has a correspondence rule given by Equation (1), where the parameter α represents the discriminatory capacity of the item; that is, how well it differentiates between examinees who have a latent trait greater than difficulty μ and those who have an ability less than μ. In this case, the graphs of two items differ not only by the displacement produced by the difficulty parameter μ but also by the function’s increasing rate, which is proportional to the parameter α (see Figure 1).

After the CAT system poses the first item to the examinee and obtains the answer, then the next step selects a second item within the CIP with characteristics depending on whether the answer is correct or incorrect. After answering the second item, the system selects the third item depending on the answers given to the first two items, whose response’s configurations are in the following set:

{(correct,correct),(correct,incorrect),(incorrect,correct),(incorrect,incorrect)},

and so on until the evaluation process of an examinee ends.

When an examinee has answered *n* items, the number of configurations of *n* Bernoulli-like trials that are elements in the derived set is $2^{n}$. In each case, a *sui generis* trajectory leads to the estimated value of the latent trait θ associated with the specific examinee. Naturally, in this case, the items are dichotomous.

One of the main characteristics of a CAT is that the test should have the smallest possible number of items and still estimate the value of the specific ability, i.e., the selection of the *n* items in a particular sequence is not arbitrary. Given the response sequence for the first n−1 items, it is possible to estimate the (n−1)-th value of the latent trait θ, which has the symbolic representation $\theta_{n-1}$.

By knowing this estimate of the latent trait at iteration (n−1)-th, the CIP provides the next most informative item [8,34,35]. Fortunately, the Fisher’s information index gives a criterion to select the most informative one (see Equation (2)),

(2) $$I\left(\theta \right)=\frac{{\left(\frac{d}{d\theta }P\left(\theta |\vec{p}\right)\right)}^{2}}{P\left(\theta |\vec{p}\right)Q\left(\theta |\vec{p}\right)},$$

where $\vec{p}$ is the vector of parameters defining the structure of the latent trait model correspondence rule and $Q\left(\theta |\vec{p}\right)=1-P\left(\theta \right|\vec{p}$). For the 1PL and 2PL models, I(θ) is given by Equations (3) and (4), respectively.

(3) $$\begin{array}{cccc} I(\theta ) & =P(\theta |\mu )Q(\theta |\mu ), & \; & \end{array}$$

(4) $$\begin{array}{ccc} \;\;I(\theta ) & =\alpha P(\theta |\mu ,\alpha )Q(\theta |\mu ,\alpha ). & \; \end{array}$$

Under the condition of independence and identical distribution of the items in the CIP, it is possible to build a likelihood function with the first (n−1) ICCs that the CAT system has applied up to the current answered items. In the best case, this likelihood function will have extreme points in the domain given by the latent feature values, implying that the likelihood function has at least one maximum [20,36].

However, the worst-case scenario is that all the first (n−1) items have a correct answer or all have a wrong answer. If one of these situations occurs, then building a likelihood function with a maximum, at least, is impossible. How does one determine the estimate of the latent trait value, in this case, to continue with the adaptive testing process?

There are several solution proposals to this problem, but a natural one [37] involves statistical information before the start of the evaluation process by using a Bayesian procedure. The idea is to use a prior distribution with which it is possible to use Bayesian argumentation to obtain estimates of the latent trait. Algorithm 1 provides a simple outline of this process.
**Algorithm 1 **Outline of the computerized adaptive assessment process.1:**procedure** EvaluationProcess2:    ${\mathrm{item}}_{1}\leftarrow \mathrm{select}\;\mathrm{the}\;\mathrm{first}\;\mathrm{item}\;\mathrm{with}\;\mathrm{parameters}\;{\vec{p}}_{1}$3:*top*: response←replytoitemi−th,1≤i4:    θ←estimatelatenttraitbasedonthepatternoffirstresponsestotheiitems5:    **if** pattern is all correct or incorrect **then**6:        **return** use *prior distribution*  and Bayes7:    **else**8:        **return** use Maximum Likelihood estimation9:      $item_{i+1}\leftarrow \mathrm{select}\;\mathrm{item}\;\mathrm{with}\;a\;\mathrm{higher}\;\mathrm{Fisher}\;\mathrm{information}\;\mathrm{in}\;\mathrm{the}\;\mathrm{Items}\;\mathrm{Pool}$10:    **goto** *top*

Note that the selection of the first item in step 2 of Algorithm 1 can proceed in at least one of two possible manners, namely

1. To calculate an estimate of the latent trait θ before starting the evaluation process and, with this estimate, to determine the item with the maximum Fisher information within the CIP [37].
2. To compute an estimate of the parameters of the first item (difficulty, discriminant, guessing, etc.) following some of the methods in [37].

Step 5 is central to the Algorithm 1 since Bayes’ Theorem requires a *prior* distribution.

Bayes’ theorem involves the use of a prior distribution to calculate the so-called posterior distribution. However, selecting an *a priori* distribution is not trivial, and one must ensure that this distribution provides the highest amount of information about each of the examinees.

The following steps are essential to the understanding of our methodology:

1. To know the relationships among the *a posteriori* probabilities, the prior probability and the likelihood function.
2. Find the *a priori* probability and its closest dependence on an academic framework.
3. The analysis of discrete and continuous cases (the latter being of greater interest).

Regarding the first step, and given in Equation (5),

(5) $$p\left(\theta |\vec{p}\right)=\frac{L(\vec{p}|\theta )}{p(\vec{p})}\cdot p\left(\theta \right),$$

we note that the likelihood function $L(\vec{p}|\theta )$ is the product of the item characteristic curves that arise throughout a specific individual evaluation pattern result. In this case, p(θ) directly gives the prior distribution.

Thus, the prior distribution is a function of the latent ability or trait θ. Finally, the transition from the discrete case to the continuous one is provided by:

(6) $$\begin{array}{ccc} S(p) & =-\int_{-\infty }^{+\infty }p\left(x\right)\log p(x)dx, & \; \end{array}$$

which may be subject to constraints of the form

(7) $$\begin{array}{ccc} \int_{-\infty }^{+\infty }f\left(x\right)p\left(x\right)dx & =\langle f(X)\rangle , & \; \end{array}$$

where 〈f(X)〉 is the expectation of the random variable defined as f(X), where *X* is a random variable whose values $x^{\prime }$s define the population of interest. Equation (7) provides the general form of the constraints.

At this stage of the CAT process, the prior distribution p(θ) and the likelihood function $L(\vec{p}|\theta )$ are available to compute the posterior distribution $p(\theta |\vec{u})$ through Equation (5). Once one calculates the posterior distribution, then estimates the next latent trait value, there are two possibilities:

- Determine the extreme point in the domain of the posterior distribution and compute the maximum value of the distribution at this extreme value.
- Determine the mean value of the latent trait population along the whole domain of the posterior distribution.

In order to sketch how the informative prior distribution can be related to the academic framework of the examinees, we propose several ansatzes.

### Ansatz for Different Indices of Student Performance as a Function of the Ability θ

Some works use the concept of entropy [38] as an alternative for the construction of informative prior distributions. In this paper we introduce the maximum entropy through the application of optimization techniques to maximize the information that the entropy will yield concerning the specific examinee.

In addition to the distribution normality constraint, the latent trait mean and variance specifications, we analyze the contribution of special examinees’ academic performance constraints to properly determine the population distribution through entropy maximization. In this sense, we apply the concept of an index (a random variable), depending on the ability θ.

By defining entropy as a cost function, entropy maximization considers that this function is subject to a list of constraints other than the constraints based on normality and first and second moments. The additional elements of the list of restrictions include the dropout rate, the failure rate, and the habits of study rates from one or more courses belonging to an examinee’s record. Additionally, one can consider the index of understanding of topics that an examinee has in a historical academic record.

To relate study habits rate and its relationship to an ability function, several authors [39,40,41,42,43] have identified some factors between good habits and excellent academic achievement:

1. Attend classes regularly.
2. Take notes while teaching.
3. Concentrate on studying.
4. Study with a view to gaining meaning, not storing facts.
5. Prepare a schedule.
6. Follow the schedule.
7. Have appropriate rest periods.
8. Facing problems considering the home environment and planning.
9. Facing the challenges posed by the school environment.
10. Keep a daily update of the work done.

**Figure 2:**
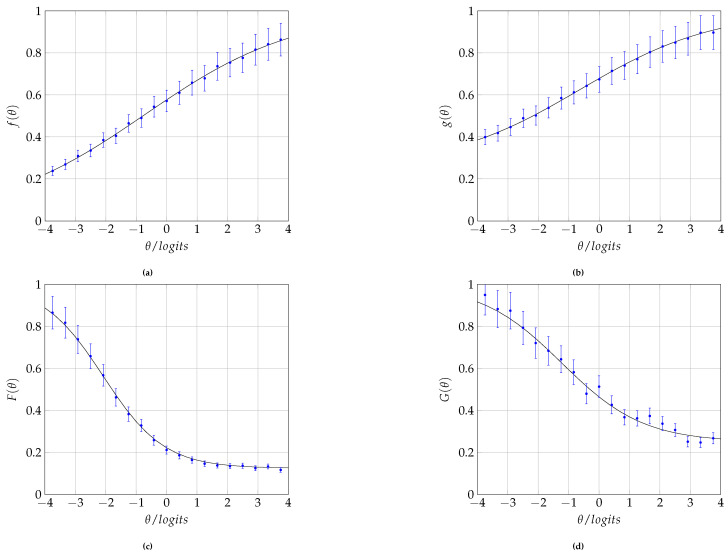
Assumed behavior for some of the indices that can be included as constraints for the determination of the prior distribution by means of entropy. (**a**) Study habit index *f* as a function of ability θ. (**b**) Subject comprehension index *g* as a function of ability θ. (**c**) Course dropout rate *F* as a function of ability θ. (**d**) Course failure rate *G* as a function of ability θ.

The statistical results in [39] confirm that the study habits index is indeed an increasing function of ability, as illustrated in Figure 2a. By applying methodologies such as those indicated by the authors in [44,45], one can adequately prepare a questionnaire including questions related to the preceding list.

On the other hand, a lack of academic and social skills leads to the student being unable to process the information transmitted by the instructor [46]. Then, we can infer that the understanding of topics is related to the student’s ability, as Figure 2b illustrates.

By means of Figure 2a, we state that the study habits index behaves sigmoidally depending on the examinee’s ability with the following correspondence rule

(8) $$f\left(\theta \right)=B_{h}+\frac{1-B_{h}}{1+b_{h}e^{-a_{h}\theta }},0<a_{h}.$$

Meanwhile, Figure 2b states that the rate of topic comprehension by students also has a sigmoidal behavior as follows

(9) $$g\left(\theta \right)=B_{c}+\frac{1-B_{c}}{1+b_{c}e^{-a_{c}\theta }},0<a_{c}.$$

In order to be rational, we consider the good study habits rate in conjunction with the students’ failure rate as a function of the ability θ as follows:

1. For a randomly selected group of students, determine their abilities $\theta_{1},\theta_{2},\ldots ,\theta_{n}$.
2. For each of the selected examinees, as indicated in the former point 1, investigate the total number of failed subjects throughout their academic history.
3. With the assigned ability, the quotient of the total number of failed subjects and the total number of subjects taken or studied (considering even repetitions or recursing) define the failure rate for a specific examinee.

The third step is reinforced by the results published in [47], where they claim that low levels of ability tend to cause dropout from a course, if not from the school itself. The failure rate has an identical behavior and, for all these reasons, Figure 2c and Figure 2d, respectively, postulate that the dropout (F(θ)) and failure (G(θ)) from a course decrease exponentially with the ability of the student. The following correspondence rules illustrate these functions:

(10) $$F\left(\theta \right)=1-\frac{1-B_{d}}{1+b_{d}e^{-a_{d}\theta }},0<a_{d},$$

(11) $$G\left(\theta \right)=1-\frac{1-B_{r}}{1+b_{r}e^{-a_{r}\theta }},0<a_{r}.$$

In all cases, note that the ability θ is a random variable and that the functions f,g,F, and *G* are, therefore, random variables. In summary, the graphs in Figure 2 are the results of ansatzes here proposed to illustrate the behaviors of the random variables *f*, *g*, *F*, and *G*.

Taking into account the postulated index functions and Equations (6) and (7), the Lagrangian L to optimize, is given by Equation (12).

(12) $$\begin{array}{cccc} L(p(\cdot )) & =-\int_{-\infty }^{+\infty }p\left(\theta \right)\log p(\theta )d\theta +\lambda_{0}\left(\int_{-\infty }^{+\infty }p\left(\theta \right)d\theta -1\right)+\lambda_{1}\left(\int_{-\infty }^{+\infty }\theta p\left(\theta \right)d\theta -{\hat{\theta }}_{p}\right) & \; & \\ \; & +\lambda_{2}\left(\int_{-\infty }^{+\infty }{\left(\theta -{\hat{\theta }}_{p}\right)}^{2}p\left(\theta \right)d\theta -{\hat{\sigma }}_{p}^{2}\right)+\lambda_{3}\left(\int_{-\infty }^{+\infty }\left(B_{h}+\frac{1-B_{h}}{1+b_{h}e^{-a_{h}\theta }}\right)p\left(\theta \right)d\theta -C_{h}\right) & \; & \\ \; & +\lambda_{4}\left(\int_{-\infty }^{+\infty }\left(B_{c}+\frac{1-B_{c}}{1+b_{c}e^{-a_{c}\theta }}\right)p\left(\theta \right)d\theta -C_{c}\right)+\lambda_{5}\left(\int_{-\infty }^{+\infty }\left(1-\frac{1-B_{d}}{1+b_{d}e^{-a_{d}\theta }}\right)p\left(\theta \right)d\theta -C_{d}\right) & \; & \\ \; & +\lambda_{6}\left(\int_{-\infty }^{+\infty }\left(1-\frac{1-B_{r}}{1+b_{r}e^{-a_{r}\theta }}\right)p\left(\theta \right)d\theta -C_{r}\right),\; & \; & \end{array}$$

where Table A1 in Appendix A provides the meaning of the symbols appearing in the equations.

Without loss of generality and for reasons of simplicity, one only considers the constraint referring to the course failure rate G(θ) so that the maximization of entropy solves the set of non-linear equations defined by Equations (13)–(16) through:

(13) $$\begin{array}{cccc} \int_{-\infty }^{+\infty }e^{-1+\lambda_{0}+\lambda_{1}\theta +\lambda_{2}{\left(\theta -{\hat{\theta }}_{p}\right)}^{2}+\lambda_{6}\left(1-\frac{1-B_{r}}{1+b_{r}e^{-a_{r}\theta }}\right)}d\theta -1 & =0, & \; & \end{array}$$

(14) $$\begin{array}{cccc} \int_{-\infty }^{+\infty }\theta e^{-1+\lambda_{0}+\lambda_{1}\theta +\lambda_{2}{\left(\theta -{\hat{\theta }}_{p}\right)}^{2}+\lambda_{6}\left(1-\frac{1-B_{r}}{1+b_{r}e^{-a_{r}\theta }}\right)}d\theta -{\hat{\theta }}_{p} & =0, & \; & \end{array}$$

(15) $$\begin{array}{cccc} \int_{-\infty }^{+\infty }{\left(\theta -{\hat{\theta }}_{p}\right)}^{2}e^{-1+\lambda_{0}+\lambda_{1}\theta +\lambda_{2}{\left(\theta -{\hat{\theta }}_{p}\right)}^{2}+\lambda_{6}\left(1-\frac{1-B_{r}}{1+b_{r}e^{-a_{r}\theta }}\right)}d\theta -{\hat{\sigma }}_{p}^{2} & =0, & \; & \end{array}$$

(16) $$\begin{array}{ccc} \int_{-\infty }^{+\infty }\left(1-\frac{1-B_{r}}{1+b_{r}e^{-a_{r}\theta }}\right)e^{-1+\lambda_{0}+\lambda_{1}\theta +\lambda_{2}{\left(\theta -{\hat{\theta }}_{p}\right)}^{2}+\lambda_{7}\left(1-\frac{1-B_{r}}{1+b_{r}e^{-a_{r}\theta }}\right)}d\theta -C_{r} & =0. & \; \end{array}$$

Note that Equation (6), both for the discrete (with summatory symbol instead of integral) and continuous cases can be considered as a measure of the misinformation (un-informativeness) that the prior distribution p(θ) provides about how the latent trait θ distributes [38,48]. This result is also supported by a research paper [48] where they state that the entropy maximization due to the constrained non-uniform prior distribution being equivalent to minimizing the distance between this distribution and an unconstrained uniform a priori distribution with no other constraint than a normalization process.

## 5. Results

Algorithm 2 illustrates our general procedure to select the a priori distribution. Line 1 of the algorithm assigns an initial estimation of the latent trait average ${\hat{\theta }}_{p}$ and variance average ${\hat{\sigma }}_{p}^{2}$. Additionally, the performance index function structures are defined as closely related to the experimental procedures (academic) already mentioned. Finally, lines 5 to 8 find the conditions to properly select an a priori distribution satisfying the normalization, ability’s average value, variance’s average value, and average values of expected performance index functions.

There are assumptions or inconveniences that appear when one applies a MAP or EAP technique at the initialization of the CAT process, considering that one does not know something about the prior distribution when the CAT system provides the first item. However, fortunately, there are several ways to solve this problem [8,35,49]. In this work, one proposes an initial ability equal to the value ${\hat{\theta }}_{p}$ given to the constraint in Equation (14). Thus we can apply Algorithm 3 to simulate the CAT process.
**Algorithm 2 **Diagram of the prior distribution search process.1:**procedure** SearchPrior2:    $\theta_{p}^{*}\leftarrow {\hat{\theta }}_{p}$                 ▹ assign average of given skill in restriction (14)3:    $\sigma_{p}^{2*}\leftarrow {\hat{\sigma }}_{p}^{2}$                   ▹ assigns expected variance in constraint (15)4:    $\vec{p}\leftarrow \mathrm{defines}\;\mathrm{index}\;\mathrm{parameters}\;\mathrm{used}\;\mathrm{in}\;\mathrm{constraints}$5:*top*:6:    s←solvesystemofnonlinearequationsdefinedbyconstraints7:    **if** unsatisfied constraints **then**8:        **goto** *top*9:    **return** $\vec{l}$                            ▹ returns Lagrange multipliers

**Algorithm 3 **Scheme of the CAT process using Bayesian estimation with prior distribution.
1:
**procedure** AdaptableEvaluationProcessWithPrior
2:    responses←[]3:    $\theta^{*}\leftarrow {\hat{\theta }}_{p}$                   ▹ allocates average of given skill in constraint (14)4:    p(θ)←determinepriordistributionmaximizingentropywithconstraints5:*top*:6:    $i\leftarrow \max_{Pool}\left\{I\left(\theta^{*}\right)\right\}$7:    r←replytoitemi,1≤i8:    responses←concat(responses,r)                 ▹ update response history9:    $L(\vec{p}|\theta )\leftarrow \mathrm{determines}\;\mathrm{the}\;\mathrm{likelihood}\;\mathrm{function}\;\mathrm{as}\;a\;\mathrm{product}\;\mathrm{of}\;\mathrm{ICCs}$10:    **if** *responses* are all correct or incorrect **then**             ▹ uses Bayesian inference11:        $p\left(\theta |\vec{p}\right)\leftarrow L\left(\vec{p}|\theta \right)p\left(\theta \right)$12:        $p\left(\theta |\vec{p}\right)\leftarrow kp\left(\theta |\vec{p}\right)$                   ▹ normalizes *posterior* distribution13:        $\theta^{*}\leftarrow \int_{-\infty }^{+\infty }\theta p\left(\theta |\vec{p}\right)d\theta$              ▹ compute average skill with new distribution14:        $p\left(\theta \right)\leftarrow p\left(\theta |\vec{p}\right)$15:    **else**16:        $\theta^{*}\leftarrow \mathrm{use}\;\mathrm{Maximum}\;\mathrm{Likelihood}\;\mathrm{estimation}\;\mathrm{as}\;\mathrm{usual}$17:    **goto** *top*.18:    **return** $\theta^{*}$


**Figure 3:**
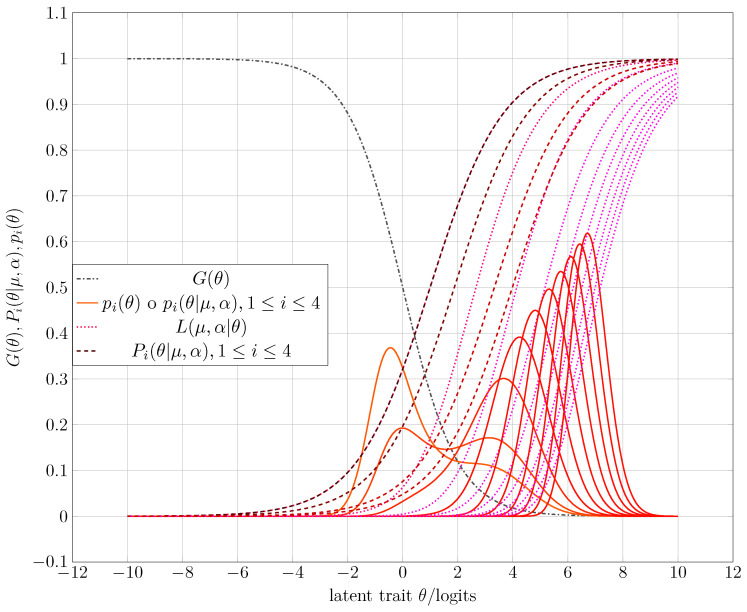
Starting of the CAT for the first experiment. Table 1 shows the expected and computed parameters from entropy maximization and the simulation process.

**Figure 4:**
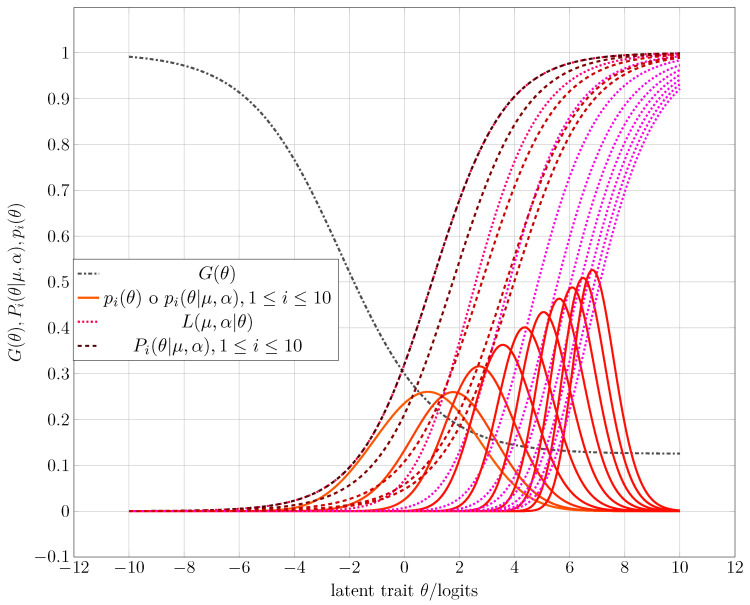
Starting of the CAT for the second experiment. Table 1 shows the expected and computed parameters from entropy maximization and the simulation process.

**Figure 5:**
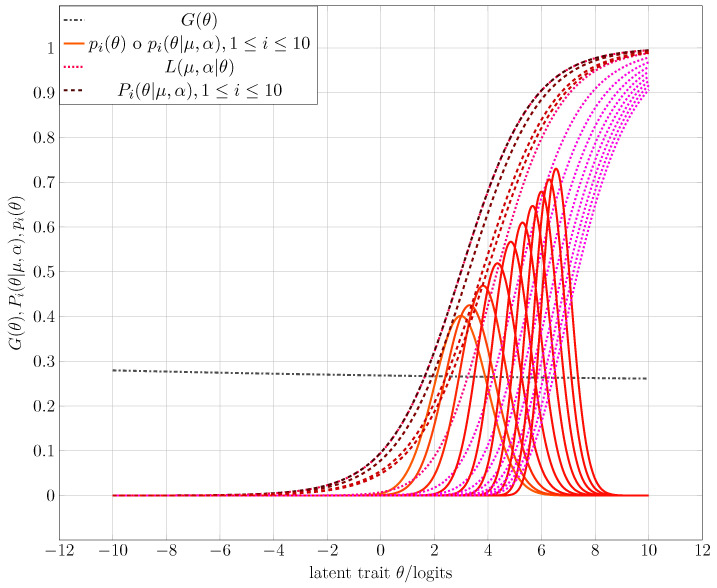
Starting of the CAT for the third experiment. Table 1 shows the expected and computed parameters from entropy maximization and the simulation process.

After running a sequence of simulations under the directions of Algorithm 3, one obtains as examples the corresponding CAT processes that Figure 3, Figure 4 and Figure 5 show. Table 1 gives some numerical results, whereas the third experiment shows the complete running. The following list synthesizes the obtained results of the corresponding simulation process.

1. After several iterations, the CAT system always tends to the maximum Fisher’s information index, regardless of the intermediate value of the estimated ability θ. Thus, the final selected item has a difficulty μ (see Figure 3, Figure 4 and Figure 5).
2. When the study habits index function discriminates well and plays the role of one constraint in entropy maximization, one can expect a bimodal a priori distribution as acceptable (see Figure 3)
3. A possible behavior in the initialization of the CAT process when the discriminating power of the study habits index function is not high or low can be found in Figure 4. Note that the a priori distribution shows some non-null skewness.
4. A failure rate with a lower discrimination index provides an initial prior distribution with almost null skewness. So, in practice, when one takes a normal or Gaussian prior distribution N(θ;μ,σ) with a high variance $\sigma^{2}$ [50] or a uniform distribution U(a,b),a≪b, one also assumes that the examinees’ failure records are the same.

There are several fine details to work out when one uses a priori distributions [51]; however, in this paper, we provide a unified approach to derive prior distributions with a less subjective selection of the distribution when the initialization of the CAT process uses Bayesian estimation [52,53].

There is a large number of research papers published about the advantages and drawbacks on the use of a priori distributions topic, but the techniques used there are based on heuristics to build the Bayesian inference procedure within the initialization of the CAT process in some special cases [51,54].

In order to compare likely differences between the results of heuristic techniques and our methodology, a useful tool to be used is the Kullback–Leibler (KL) divergence index. This index measures the divergence of the expected amount of extra information required to obtain population samples that follow the prior distribution p(θ) when using population samples that follow a distribution q(θ) [55].

The KL divergence measure is defined by Equation (17).

(17) $$\begin{array}{ccc} D_{KL}(p\left(\theta \right)||q\left(\theta \right) & =\int_{-\infty }^{+\infty }p\left(\theta \right)\ln\frac{p(\theta )}{q(\theta )}d\theta . & \; \end{array}$$

In this sense, the information is more ordered when one applies the prior distribution obtained with our method than with the popular unconstrained heuristic distributions. One should expect this result since the introduction of constraints orders the information under analysis. In this manner, p(θ) represents a “realistic” data distribution or a precisely calculated theoretical distribution and the typical distribution q(θ) represents a description or approximation of p(θ) (see [55]).

Through Table 1, a correspondence rule for the a priori distribution p(θ) can be defined such as Table 2 illustrates. Therefore, the two measures given by Equations (18) and (19) considering the normal and uniform distributions, respectively, can be calculated.

(18) $$\begin{array}{cccc} D_{KL}\left(p\left(\theta \right)||N\left(\theta ;\mu ,\sigma \right)\right) & =\int_{-\infty }^{+\infty }p\left(\theta \right)\ln\frac{p(\theta )}{N(\theta ;\mu ,\sigma )}d\theta , & \; & \end{array}$$

(19) $$\begin{array}{ccc} D_{KL}\left(p\left(\theta \right)||U\left(\theta ;a,b\right)\right) & =\int_{-\infty }^{+\infty }p\left(\theta \right)\ln\frac{p(\theta )}{U(\theta ;a,b)}d\theta . & \; \end{array}$$

**Figure 6:**
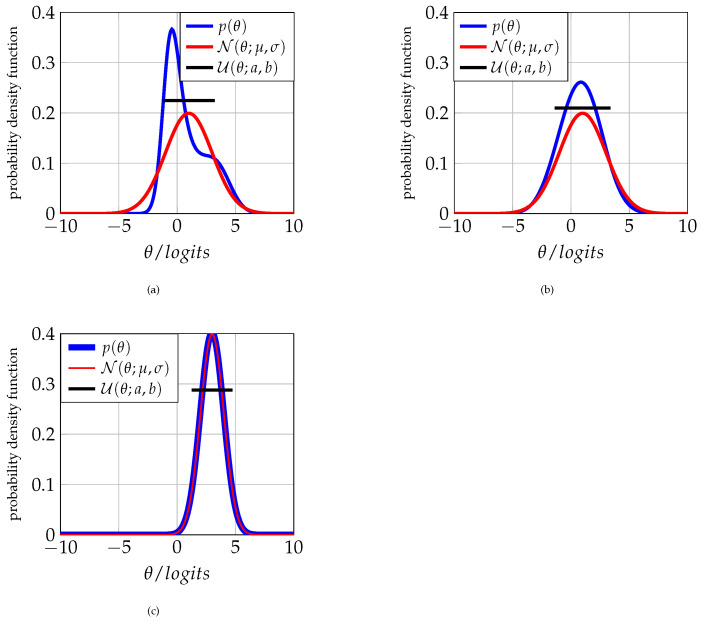
KL distance between each of the initial prior distributions p(θ) from each experiment and the classic distributions N(θ;μ,σ) and U(θ;a,b). (**a**) First experiment: p(θ) vs. N(θ;1,2) and U(θ;a,b). (**b**) Second experiment: p(θ) vs. N(θ;1,2) and U(θ;a,b). (**c**) Third experiment: p(θ) vs. N(θ;3,1) and U(θ;a,b).

From Table 2, note that the distance given by the KL divergence in the first experiment when comparing the prior distribution with the Gaussian distribution N(θ;μ,σ) suggests that to analyze the population with this last distribution, one should expect an amount of 3.5402 extra information to include the data population related to the first distribution. Figure 6a–c compare the three distributions for every experiment in Table 2, and show their respective KL measures.

In the first experiment, when one compares the distribution U(θ;1−2.225,1+2.225), the a priori distribution p(θ) also has a KL divergence equal to 0.73339. On the other hand, for the second experiment, KL divergences become equal to 2.4119 and 0.077474 when one, respectively, approximates through N(θ;1,2) and U(θ,1−2.385,1+2.385). Finally, for the third experiment, the KL divergences equal to 0.069902 and 0.084054 when one, respectively, approximates through N(θ;3,1) and U(θ;3−1.7375,3+1.7375).

Intervals (a,b) for every uniform distribution are calculated by looking for the lower distance between the corresponding p(θ) and U(θ;a,b) distributions. Note that the third experiment results agree with the heuristic suggestion of using the normal, or uniform distributions, as good approximations to the prior distribution. So, the alternative is acceptable when the course failure index function does not discriminate well.

## 6. Conclusions

In this paper, we demonstrate that through the theory of entropy maximization, a given set of constraints, and under numerical experimentation, the computation of an a priori distribution to initialize a CAT process by using Bayesian inference can be carried out. Furthermore, the examinee’s performance index functions define the constraints, and they complement the usual distribution constraints (normality, first and second moment, etc.).

We also show that through the entropy theory, the selection of appropriate constraints summarizes experimental data through the specification of index functions related to study habits, comprehension levels, course dropout, and lecture failure.

A given set of constraints can produce a set of acceptable or unacceptable a priori distributions, so one needs to look for a stop criterion in searching for the optimal set of parameters that defines the distributions through entropy maximization. To define the stop criterion, we verify how the estimated set of distribution parameters and those that define the constraints are close enough to the expected values used in the constraints definition. Thus, the most appropriate distribution is chosen and, under the assumption of responding correctly to the first items in the testing process, we can verify its latent trait prediction capability.

Index functions playing the role of constraints with acceptable discrimination properties produce a priori distributions with bimodality, as one can expect, so that the obtained distribution estimates reasonable latent trait values along the simulation of the CAT process.

In summary, entropy maximization can be used inside the frame of a CAT to derive more generalized a priori distributions through constraint specifications related to index functions. This method can provide a unified approach to derive a priori distributions for initializing the CAT process through a Bayesian inference procedure.

## Figures and Tables

**Table 1:** Results of numerical experimentation. The parameters values of the Start Parameters column assume that they come from estimations and/or experiments. The parameters values of the prior properties column assume that they come from the computation of the corresponding integral expression containing the computed prior distribution (first terms in left-hand sides of Equations (13)–(16)). The numerical experimentation of a running for a CAT in Experiment 3 assumes the existence of a pool of 1000 calibrated items.

Experiment	Start Parameters						*prior* properties					Lagrange’s multipliers			
1	$\theta_{p}^{*}$	$\sigma_{p}^{*2}$	$a_{r}$	$b_{r}$	$B_{r}$	$C_{r}^{*}$	normality	θ	$\sigma^{2}$	$C_{r}$	skewness	$\lambda_{1}$	$\lambda_{2}$	$\lambda_{3}$	$\lambda_{4}$
	1	4	1	1	0	0.6	1.18	0.98	3.99	0.47	3.66	−6.60	2.12	−0.40	13.74
Experiment	Start Parameters						Properties *prior*					Lagrange’s multipliers			
2	$\theta_{p}^{*}$	$\sigma_{p}^{*2}$	$a_{r}$	$b_{r}$	$B_{r}$	$C_{r}^{*}$	normality	θ	$\sigma^{2}$	$C_{r}$	skewness	$\lambda_{1}$	$\lambda_{2}$	$\lambda_{3}$	$\lambda_{4}$
	1	4	0.6	0.25	0.125	0.6	1.20	0.96	3.99	0.34	−2.00	−1.34	0.16	−0.19	3.62
Experiment	Start Parameters						Properties *prior*					Lagrange’s multipliers			
3	$\theta_{p}^{*}$	$\sigma_{p}^{*2}$	$a_{r}$	$b_{r}$	$B_{r}$	$C_{r}^{*}$	normality	θ	$\sigma^{2}$	$C_{r}$	skewness	$\lambda_{1}$	$\lambda_{2}$	$\lambda_{3}$	$\lambda_{4}$
	3	1	0.05	0.025	0.25	0.3	1.00	3.00	1.00	0.27	−0.04	−3.49	0.00	−0.5	13.46
	1000 difficulties in items Pool														
	iteration			$\theta_{p}^{*}$			$\mu_{p}^{*}$			normality		map		eap	
	1			3			2.9978			1.00411027		2.99		3.00159777	
	2			3.3222			3.3207			1.00		3.32		3.32218745	
	3			3.8357			3.8430			1.00		3.82		3.83572950	
	4			4.3893			3.9851			1.00		4.36		4.38926317	
	5			4.8986			3.9851			1.00		4.85		4.89863112	
	6			5.3412			3.9851			1.00		5.29		5.34123495	
	7			5.7224			3.9851			1.00		5.67		5.72237081	

**Table 2:** A priori distributions distances with respect to heuristics ones for every experiment in Table 1.

Experiment	p(θ)	q(θ)	KL Distance
1	$e^{-1+\lambda_{1}+\lambda_{2}\theta +\lambda_{3}{\left(\theta -\theta_{p}^{*}\right)}^{2}+\lambda_{4}\left(1-\frac{1-Br}{1+b_{r}e^{-a_{r}\theta }}\right)}$	N(θ;1,2)	3.5402
		U(θ;1−2.225,1+2.225)	0.73339
2	$e^{-1+\lambda_{1}+\lambda_{2}\theta +\lambda_{3}{\left(\theta -\theta_{p}^{*}\right)}^{2}+\lambda_{4}\left(1-\frac{1-Br}{1+b_{r}e^{-a_{r}\theta }}\right)}$	N(θ;1,2)	2.4119
		U(θ;1−2.385,1+2.385)	0.077474
3	$e^{-1+\lambda_{1}+\lambda_{2}\theta +\lambda_{3}{\left(\theta -\theta_{p}^{*}\right)}^{2}+\lambda_{4}\left(1-\frac{1-Br}{1+b_{r}e^{-a_{r}\theta }}\right)}$	N(θ;3,1)	0.069902
		U(θ;3−1.7375,3+1.7375)	0.084054

## Data Availability

Not applicable.

## Abbreviations

The following abbreviations are used in this manuscript:

1PL	One-Parameter Logistic
2PL	Two-Parameter Logistic
CAT	Computer Adaptive Testing
CIP	Calibrated Item Pool
EAP	Expectation a posteriori
IRT	Item Response Theory
ICC	Item Characteristic Curve
KL	Kullback–Leibler
MAP	*maximum a posteriori*
w.r.to	with respect to

## Appendix A. Table of Symbols

**Table A1:** Summary of all the symbols used in the paper, their location and meaning.

Symbol	Meaning	Pages Where the Symbol Appears
*e*	Base of the natural logarithm	2
θ	Examinee’s ability or latent trait to be estimated in the CAT process	2–6, 10, 12–16
$\theta_{i}$	Examinee’s ability at *i*-th iteration	6
μ	Item’s difficulty playing the role of a parameter in the definition of the ICC that the CDF *P* defines	2,5,6,15,16
$\mu_{i},i=1,2,\ldots$	Difficulty of item *i*	5
α	Item’s discriminant playing the role of a parameter in the definition of the ICC that the PDF *P* defines	5,6,13,14
*P*	Conditional cumulative distribution function for computing the probability that the examinee with ability θ gives a correct answer to an item, given that the item’s difficulty is μ and (possibly) the discriminant is α	2,5,6
*Q*	It is equal to 1−P; in other words, it represents the probability that the examinee with ability θ gives an incorrect answer to an item, given that the item’s difficulty is μ and (possibly) the discriminant is α	6
Δμ	Increment of item’s difficulty	6
Δα	Increment of item’s discriminant	5
n=1,2,…	Number of items at iteration *n*, or *n*-th iteration, in the CAT process	6
*I*	Fisher’s information, as a function of the examinee’s ability θ, is useful to compute the amount of information that a given item provides about the θ estimate by knowing the current value of θ	6
$\vec{p}$	Vector of parameters (item’s difficulty, item’s discriminant, etc.) that defines the model *P*	6,12
*p*	If *p* depends on θ, then it represents a prior distribution; otherwise, if *p* depends on θ given the vector of parameters $\vec{p}$, then it represents a posteriori distribution	7,16
*L*	Likelihood function depends on the vector of parameters $\vec{p}$ given the examinee’s ability θ, $L\left({\vec{p}}_{i},i=1,2,\ldots ,n|\theta \right)\propto \prod_{i=1}^{n}P_{i}^{k}\left(\theta |{\vec{p}}_{i}\right)Q_{i}^{n-k}\left(\theta |{\vec{p}}_{i}\right)$, where *k* represents the number of correct answers that an examinee gives to *n* items	7
f,g,F,G	Performance indexes are functions of a random variable *X*	7
S	Entropy	7
L	Lagrangian	8,11
λ	Lagrange multiplier	8,11
$B_{i},b_{i},a_{i}$, where i=h,c,d,r	Parameters defining performance index function: study habit, subject comprehension, course dropout rate, and course failure rate, respectively	10
${\hat{\theta }}_{p}$	Initial estimation of the latent trait θ, just before starting the CAT process, the subindex *p* comes from the word prior	11,12
${\hat{\sigma }}_{p}^{2}$	Initial estimation of the variance $\sigma^{2}$ of the distribution of the latent trait θ, the subindex *p* comes from the word *prior*	11,12
*s*	It contains the solution to entropy maximization under the given constraints	12
$\vec{l}$	Vector of Lagrange multipliers after satisfying entropy maximization under given constraints	12
$\theta^{*}$	Initial estimation of the latent trait θ, just before starting the CAT process, or expectation a posteriori of the latent trait along the CAT process, or the extreme point of the ability values θ, along the CAT process, where the a posteriori distribution has a *maximum*	12,13
U	Uniform distribution	13,15,16
N	Normal distribution	13,15,16
a,b with a<b	lower and upper bounds, respectively, that define the uniform distribution	15,16
$\sigma^{2},\sigma$	Variance and standard deviation estimation, respectively, for a priori distribution	13,15
$C_{r}$	Estimation of the course failure rate average by means of the estimated a priori distribution	11,13
$C_{r}^{*}$	Course failure rate estimation coming from the experimental results for obtaining the course failure rate index function	11,13
